# Merlin^S13^ phosphorylation controls meningioma Wnt signaling and magnetic resonance imaging features

**DOI:** 10.21203/rs.3.rs-2577844/v1

**Published:** 2023-03-14

**Authors:** Charlotte Eaton, Lauro Avalos, S. John Liu, Tim Casey-clyde, Paola Bisignano, Calixto-Hope Lucas, Erica Stevenson, Abrar Choudhury, Harish Vasudevan, Stephen Magill, Nevan Krogan, Javier Villanueva-Meyer, Danielle Swaney, David Raleigh

**Affiliations:** University of California San Francisco; University of California San Francisco; University of California San Francisco; University of California San Francisco; Vanderbilt; Johns Hopkins University; University of California, San Francisco; University of California, San Francisco; University of California San Francisco; Northwestern University; Quantitative Biosciences Institute, University of California San Francisco; UCSF; UCSF; University of California San Francisco

## Abstract

Meningiomas are the most common primary intracranial tumors and are associated with inactivation of the tumor suppressor *NF2*/Merlin, but one-third of meningiomas retain Merlin expression and typically have favorable clinical outcomes. Biochemical mechanisms underlying Merlin-intact meningioma growth are incompletely understood, and non-invasive biomarkers that predict meningioma outcomes and could be used to guide treatment de-escalation or imaging surveillance of Merlin-intact meningiomas are lacking. Here we integrate single-cell RNA sequencing, proximity-labeling proteomic mass spectrometry, mechanistic and functional approaches, and magnetic resonance imaging (MRI) across meningioma cells, xenografts, and human patients to define biochemical mechanisms and an imaging biomarker that distinguish Merlin-intact meningiomas with favorable clinical outcomes from meningiomas with unfavorable clinical outcomes. We find Merlin drives meningioma Wnt signaling and tumor growth through a feed-forward mechanism that requires Merlin dephosphorylation on serine 13 (S13) to attenuate inhibitory interactions with β-catenin and activate the Wnt pathway. Meningioma MRI analyses of xenografts and human patients show Merlin-intact meningiomas with S13 phosphorylation and favorable clinical outcomes are associated with high apparent diffusion coefficient (ADC) on diffusion-weighted imaging. In sum, our results shed light on Merlin posttranslational modifications that regulate meningioma Wnt signaling and tumor growth in tumors without *NF2*/Merlin inactivation. To translate these findings to clinical practice, we establish a non-invasive imaging biomarker that could be used to guide treatment de-escalation or imaging surveillance for patients with favorable meningiomas.

## Introduction

Meningiomas arising from the meningothelial lining of the central nervous system comprise over 40% of primary intracranial tumors^1,2^, and approximately 1% of humans will develop a meningioma in their lifetime^3^. Meningiomas are treated with surgery and radiotherapy, and systemic therapies remain ineffective or experimental for patients with meningiomas^4^. Bioinformatic investigations have revealed biological drivers and therapeutic vulnerabilities underlying meningiomas with unfavorable outcomes^5-12^, and clinical trials of new therapeutic strategies to treat patients with meningiomas that are resistant to standard interventions are underway^4^. Nevertheless, most meningiomas are benign and many can be safely observed with serial magnetic resonance imaging (MRI)^13^. Imaging surveillance can spare patients from morbidities associated with potentially unnecessary surgical or radiotherapy treatments, but current meningioma classification systems rely on histological and molecular analyses of tumor tissue after resection^14^. Thus, there is an unmet need to develop non-invasive imaging biomarkers that predict meningioma outcomes and could be used to guide treatment de-escalation or surveillance for the most common primary intracranial tumor.

Meningiomas are often associated with inactivation of the tumor suppressor *NF2*/Merlin^15^, but approximately one-third of meningiomas are Merlin-intact and have favorable clinical outcomes^7-9,11,16,17^. Merlin is a member of the FERM family of proteins (Four-point-one, Ezrin, Radixin, and Moesin) that link the cytoskeleton to the plasma membrane and are comprised of a FERM domain, an α-helical domain, and a C-terminal domain (CTD). Merlin-intact meningiomas can encode somatic short variants (SSVs) targeting *TRAF7, PIK3CA, AKT1, KLF4,* or the Hedgehog pathway^16,17^, but some of these variants may be passenger mutations that do not influence meningioma tumorigenesis^18-20^. These data suggest biochemical mechanisms driving Merlin-intact meningiomas are incompletely understood. Here we test the hypothesis that understanding signaling mechanisms in Merlin-intact meningiomas may shed light on new strategies to define meningioma biology pre-operatively using non-invasive imaging techniques.

## Results

**Figure 1:**
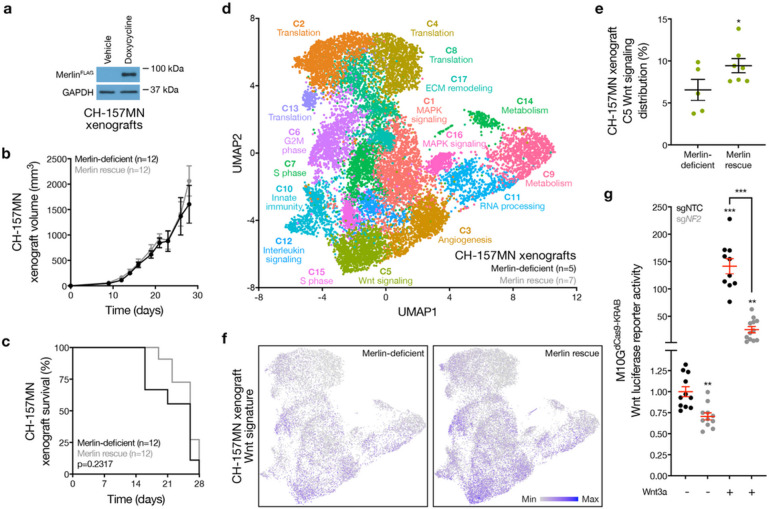
Merlin drives meningioma Wnt signaling. **a**, Immunoblots for FLAG (Merlin) or GAPDH in CH-157MN meningioma xenografts with or without 24-hours of doxycycline-inducible Merlin rescue (200μg/ml). **b**, CH-157MN xenograft measurements in NU/NU mice with (n=12) or without (n=12) doxycycline-inducible Merlin rescue as in **a**. **c**, Kaplan-Meier survival curve for CH-157MN xenograft overall survival in NU/NU mice as in **b** (log-rank test). **d**, Uniform manifold approximation and projection (UMAP) of single-cell RNA sequencing transcriptomes of 40,765 CH-157 cells from 12 xenografts as in **a-c** colored by assignments from Louvain clustering. **e,** Quantification of single-cell types from **d** that were differentially enriched in Merlin-deficient compared to Merlin rescue xenografts. **f,** Feature plots for the MSigDB Hallmark Wnt target gene expression signature in single-cell transcriptomes from Merlin-deficient compared to Merlin rescue xenografts. **g,** TOP-Flash Tcf/Lef luciferase reporter assay in M10G^dCas9-KRAB^ meningioma cells expressing non-targeted control sgRNAs (sgNTC) or sgRNAs suppressing *NF2* (sg*NF2*) with or without 24-hours of Wnt3a treatment (100ng/μl). Lines represent means, and error bar represent standard error of the means. *P≤0.05, **P≤0.01, ***P≤0.0001 (Student’s t-test, one sided).

To study Merlin signaling mechanisms in meningiomas, CH-157MN human meningioma cells lacking endogenous Merlin^21^ were transduced with a doxycycline-inducible *NF2* construct and grown as xenografts in mice (Fig. 1a). Merlin rescue with doxycycline in CH-157MN cells did not influence meningioma histology, growth, or overall survival compared to xenografts in mice without doxycycline (Fig. 1b, c and Extended Data Fig. 1). To validate these findings, IOMM-Lee human meningioma cells encoding endogenous Merlin^22^ were transduced with short-hairpin RNAs (shRNAs) suppressing *NF2* (sh*NF2*), or non-targeted control shRNAs (shNTC), and grown as xenografts in mice (Extended Data Fig. 2a). Merlin loss in IOMM-Lee cells did not influence meningioma histology, growth, or overall survival compared to mice with IOMM-Lee cells expressing shNTC (Extended Data Fig. 2b-e). Single-cell RNA sequencing was performed on 40,765 human meningioma cells from 5 Merlin-deficient CH-157MN xenografts and 7 CH-157MN xenografts with Merlin rescue (Extended Data Fig. 3a). Uniform manifold approximation projection (UMAP) and Louvain clustering defined 17 meningioma xenograft cell states (Fig. 1d), which were distinguished using differentially expressed genes and phases of the cell cycle (Extended Data Fig. 3b, c and Supplementary Table 1). Comparison of meningioma cell states across xenografts revealed only 1 cluster was enriched in Merlin rescue xenografts compared to Merlin deficient xenografts (Fig. 1e). This cluster was distinguished by expression of the Wnt pathway effector *CTNNB1*/β-catenin (Extended Data Fig. 3a). In support of these data, Wnt target genes were also enriched in single cells from Merlin rescue xenografts compared to Merlin-deficient xenografts (Fig. 1f).

Markers of Wnt pathway activation have been identified in human meningiomas^23^, but how Wnt signals are transduced through meningioma cells is unknown. Canonical Wnt signaling proceeds through β-catenin, which is degraded by a destruction complex that is active the absence of Wnt stimulation^24^. Wnt stimulation induces PP1A to de-phosphorylate and inactivate the β-catenin destruction complex^25^, allowing β-catenin to localize to the plasma membrane or to the nucleus in complex with the transcriptional co-activator Tcf/Lef.

To determine if Merlin regulates Wnt signaling in meningioma cells, M10G human meningioma cells stably expressing CRISPR interference (CRISPRi) machinery (dCas9-KRAB)^26,27^ were transduced with non-targeted control sgRNAs (sgNTC) or sgRNAs suppressing endogenous *NF2*(sg*NF2*) (Extended Data Fig. 4a). Co-transfection of the TOP-Flash Tcf/Lef Wnt luciferase reporter and treatment with recombinant Wnt3a or vehicle control revealed Merlin suppression attenuated Wnt signaling in meningioma cells (Fig. 1g). Beyond meningioma, *NF2*/Merlin inactivation is also associated with schwannoma tumorigenesis^15^, and Merlin suppression with sg*NF2* in human HEI-193 schwannoma cells stably expressing CRISPRi machinery also attenuated Wnt signaling compared to sgNTC (Extended Data Fig. 4b, c).

**Figure 2:**
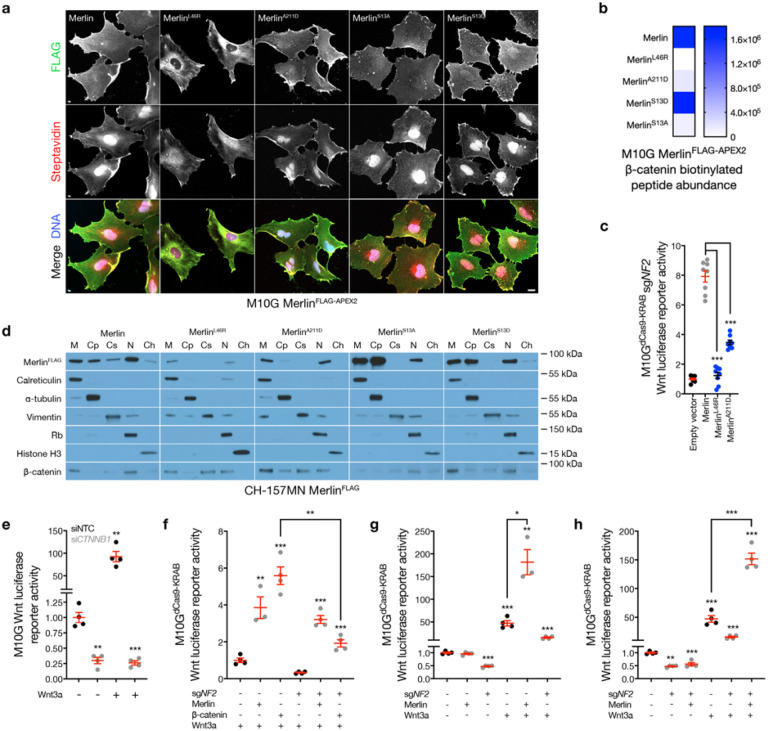
Merlin regulates b-catenin localization in meningioma cells. **a**, Immunofluorescence for FLAG (Merlin) or streptavidin in M10G meningioma cells expressing Merlin^FLAG-APEX2^ constructs after proximity-labelling. Scale bar, 10 μm. **b**, Heatmap of b-catenin biotinylation peptide intensity from proximity-labeling proteomic mass spectrometry as in **a** (n=3/condition). **c**, TOP-Flash Tcf/Lef luciferase reporter assay in M10G^dCas9-KRAB^ meningioma cells expressing sgRNAs suppressing *NF2* (sg*NF2*) with or without rescue of Merlin^FLAG-APEX2^ wildtype or cancer-associated missense constructs. **d**, Immunoblots for FLAG (Merlin) or b-catenin after biochemical fractionation of CH-157MN meningioma cells expressing Merlin^FLAG-APEX2^ rescue constructs. Immunoblots for calreticulin, a-tubulin, vimentin, Rb, or histone H3 mark membrane (M), cytoplasmic (Cp), cytoskeletal (Cs), nuclear (N), or chromatin fractions (Ch), respectively. **e**, TOP-Flash Tcf/Lef luciferase reporter assay in M10G meningioma cells expressing non-targeted control siRNAs (siNTC) or siRNAs suppressing b-catenin (si*CTNNB1*) with or without 24-hours of Wnt3a treatment (100ng/ul). **f-h**, TOP-Flash Tcf/Lef luciferase reporter assays in M10G^dCas9-KRAB^ meningioma cells expressing non-targeted control sgRNAs (sgNTC) or sg*NF2* with or without 24-hours of Wnt3a treatment (100ng/μl) in the presence or absence of b-catenin or Merlin overexpression or rescue. **f** shows b-catenin overexpression fails to hyperactivate the Wnt pathway in the absence of Merlin. **g** and **h** show Wnt stimulation is necessary for Merlin overexpression to hyperactivate the Wnt pathway. Lines represent means, and error bar represent standard error of the means. **P≤0.01, ***P≤0.0001 (Student’s t-test, one sided).

To identify candidates underlying Merlin regulation of the Wnt pathway, M1OG cells were transduced with doxycycline-inducible wildtype (WT) or cancer-associated missense Merlin constructs (L46R, A211D) that encoded C-terminal FLAG and APEX2 tags to enable subcellular localization and proximity labeling proteomic mass spectrometry studies^28^. Immunofluorescence or immunoblots after biochemical subcellular fractionation demonstrated decreased stability and re-localization of Merlin^L46R^ and Merlin^A211D^ compared to Merlin^WT^ that was amplified after streptavidin labeling of biotinylated peptides in proximity to Merlin constructs (Fig. 2a and Extended Data Fig. 5a). Streptavidin pulldown and proximity-labeling proteomic mass spectrometry identified β-catenin adjacent to Merlin^WT^ but not Merlin^L46R^, and also revealed that β-catenin was diminished in proximity to Merlin^A211D^ (Fig. 2b and Supplementary Table 2). In support of these data, Merlin^L46R^ and Merlin^A211D^ were unable to rescue Wnt signaling in M10G cells with CRISPRi suppression of endogenous *NF2* (Fig. 2c). β-catenin was not degraded by loss of *NF2* (Extended Data Fig. 5b). Subcellular fractionation of CH-157MN cells after doxycycline-inducible Merlin rescue showed β-catenin was distributed across the plasma membrane, cytoplasm, cytoskeleton, and nucleus with Merlin^L46R^ and Merlin^A211D^ rescue, but was only enriched at the plasma membrane and in the nucleus with Merlin^WT^ rescue (Fig. 2d), β-catenin suppression using siRNAs (si*CTNNB1*) inhibited meningioma Wnt signaling (Fig. 2e and Extended Data Fig. 5c), but Merlin was required for maximal Wnt pathway activation in meningioma cells even after β-catenin overexpression (Fig. 2f and Extended Data Fig. 5d). In the absence of Wnt3a, Merlin overexpression did not activate the Wnt pathway with or without endogenous Merlin, but endogenous Merlin suppression attenuated Wnt signaling (Fig. 2g, h). In the presence of Wnt3a, overexpression of Merlin activated the Wnt pathway regardless of endogenous Merlin status (Fig. 2g, h). In sum, these data suggest Merlin regulates the Wnt pathway through a feed-forward mechanism that influences Merlin-β-catenin interaction and subcellular localization.

**Figure 3:**
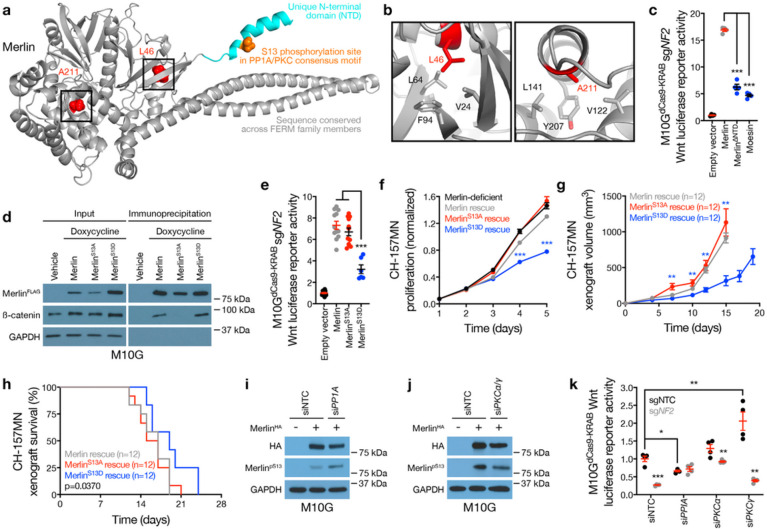
Merlin dephosphorylation at Serine 13 activates meningioma Wnt signaling. **a**, Full-length structural model of Merlin, based on the crystal template 4RZJ. Red shows cancer-associated missense mutations. Cyan shows the NTD, which is not conserved in other FERM family members, containing a consensus PP1A/PKC phosphorylation motif and phosphorylation site (S13) in orange. **b**, the 4RZJ X-ray structure of Merlin shows L46 and A211 in hydrophobic pockets that may be destabilized by charged cancer-associated missense mutations. **c**, TOP-Flash Tcf/Lef luciferase reporter assay in M10G^dCas9-KRAB^ meningioma cells expressing sgRNAs suppressing *NF2* (sg*NF2*) with or without rescue of Merlin constructs or overexpression of the FERM family member Moesin. **d**, Immunoblots for FLAG (Merlin), b-catenin, or GAPDH before versus after FLAG immunoprecipitation from M10G meningioma cells with or without doxycycline-inducible Merlin^FLAG^ overexpression (1μg/ml). **e**, TOP-Flash Tcf/Lef luciferase reporter assay in M10G^dCas9-KRAB^ meningioma cells expressing sg*NF2* with or without rescue of Merlin wildtype or S13 unphosphorylatable (S13A) or phospho-mimetic (S13D) constructs. **f**, CH-157MN meningioma cell MTT assays for cell proliferation with or without doxycycline-inducible Merlin rescue (100ng/ml). **g**, CH-157MN xenograft measurements in NU/NU mice with or without doxycycline-inducible Merlin rescue (20μg/ml) as in **f**. **h**, Kaplan-Meier survival curve for CH-157MN xenograft overall survival in NU/NU mice as in **g** (log-rank test). **i, j**, Immunoblots for HA (Merlin) or Merlin with phosphorylated S13 (Merlin^pS13^) after Merlin immunoprecipitation from M10G cells with or without Merlin overexpression and concurrent expression of non-targeted control siRNAs (siNTC) or siRNAs suppressing PP1A or PKC isoforms. GAPDH immunoblots are shown from immunoprecipitation inputs as a loading control. **k**, TOP-Flash Tcf/Lef luciferase reporter assay in M10G^dCas9-KRAB^ meningioma cells expressing non-targeted control sgRNAs (sgNTC) or sg*NF2* with concurrent siNTC or siRNA suppression of PP1A or PKC isoforms. Lines represent means, and error bar represent standard error of the means. *P≤0.05, **P≤0.01, ***P≤0.0001 (Student’s t-test, one sided).

Visual inspection of available crystal structures of Merlin (PDB 4ZRJ) revealed that L46R and A211D cancer-associated missense mutations were located on α-helices embedded in hydrophobic pockets of the Merlin FERM domain, suggesting that charged amino acid substitutions of L46 or A211 may destabilize the secondary structure of the protein (Fig. 3a, b). The Merlin N-terminal domain (NTD) is unique among FERM family members, and structural modeling showed the NTD is a flexible, 19-residue α-helix that protrudes from the surface of the protein (Fig. 3a). Overexpression of Moesin, or Merlin rescue using a construct lacking the NTD (Merlin^ΔNTD^), were unable to rescue Wnt signaling in Merlin deficient meningioma cells (Fig. 3c and Extended Data Fig. 6a). Thus, we hypothesized the Merlin NTD may regulate Merlin/β-catenin interaction and Wnt signaling in meningiomas.

Sequence analysis of the Merlin NTD showed a phosphorylation site on serine 13 (S13) within consensus motifs for a kinase (PKC) and a phosphatase (PP1A), both of which are core components of the Wnt pathway that were also identified in proximity to Merlin^FLAG-APEX2^ constructs in meningioma cells (Supplementary Table 2). Proteomic proximity-labeling mass spectrometry of unphosphorylatable (S13A) or phospho-mimetic (S13D) Merlin constructs revealed β-catenin adjacent to Merlin^S13D^ but not Merlin^S13A^ (Fig. 2a, b). Immunoprecipitation and immunoblots validated β-catenin interaction with Merlin^S13D^ but not Merlin^S13A^ (Fig. 3d). Rescue of Merlin^S13D^ but not Merlin^S13A^ sequestered β-catenin at the plasma membrane in meningioma cells (Fig. 2d), and Merlin^S13A^ but not Merlin^S13D^ rescued Wnt signaling in meningioma cells lacking *NF2*(Fig. 3e). Moreover, Merlin^S13D^ rescue attenuated meningioma cell proliferation *in vitro* (Fig. 3f), inhibited meningioma xenograft growth *in vivo* (Fig. 3g and Extended Data Fig. 6b, c), and prolonged overall survival compared to Merlin^WT^ or Merlin^S13A^ rescue (Fig. 3h). Immunoblots of meningioma cell lysates after shRNA suppression of PKC ± phosphatase inhibition revealed a Merlin doublet that was eliminated with suppression of PKCα or PKCγ, or with overexpression of Merlin^S13A^ (Extended Data Fig. 6d). A novel phospho-specific antibody recognizing Merlin^pS13^ (Extended Data Fig. 6e) showed small-interfering RNAs (siRNAs) suppressing PP1A increased Merlin^pS13^ immunoblot intensity but siRNAs suppressing PKCα and PKCγ decreased Merlin^pS13^ immunoblot intensity compared to non-targeted control siRNAs (siNTC) in meningioma cells (Fig. 3i, j and Extended Data Fig. 6f). Moreover, Merlin-dependent Wnt signaling was attenuated by siRNAs suppressing PP1A and activated by siRNAs suppressing PKCγ in meningioma cells compared to siNTC (Fig. 3k).

Surgery is the mainstay of meningioma treatment and is often essential to relieve neurological symptoms from tumor mass effect^29^. Nevertheless, many meningiomas are diagnosed incidentally or with minimal presenting symptoms, and the majority of incidentally-diagnosed meningiomas will not grow on long-term imaging surveillance^13^. These clinical observations underscore the unmet need for non-invasive, clinically-tractable biomarkers that predict meningioma outcomes and could be used to guide treatment de-escalation or imaging surveillance. Qualitative MRI features such as peritumoral edema, tumor calcification, tumor location, adjacent bone destruction, or irregular tumor margins can be associated with higher-grade meningiomas on preoperative imaging studies^30-32^. Although MRI can easily diagnose meningiomas, qualitative approaches are not reliable for distinguishing meningioma outcomes^33-36^. Quantitative apparent diffusion coefficient (ADC) hypointensity on diffusion-weighted MRI is prognostic for unfavorable meningioma outcomes^37^, and may be associated with meningioma Wnt signaling^27^, but biochemical mechanisms underlying meningioma imaging features are unknown.

**Figure 4:**
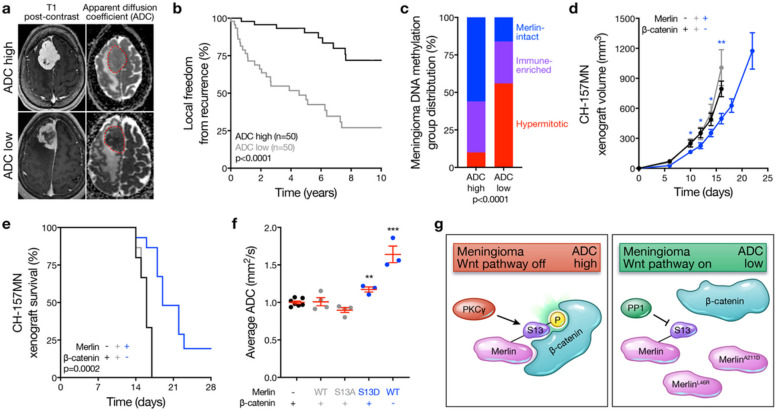
High MRI apparent diffusion coefficient distinguishes Merlin-intact meningiomas with favorable clinical outcomes. **a**, Example brain magnetic resonance imaging (MRI) T1 post-contrast and apparent diffusion coefficient (ADC) maps. Red dotted lines show meningiomas on ADC maps. **b**, Kaplan-Meier survival curve for local freedom from recurrence in 100 human meningioma patients with pre-operative MRI analysis and post-operative DNA methylation profiling dichotomized at the mean normalized ADC (1.21, log-rank test). **c**, Meningioma DNA methylation groups across ADC high versus ADC low strata from the 100 patients in **b** (Chi-squared test). **d**, CH-157MN xenograft measurements in NU/NU mice with or without doxycycline-inducible Merlin rescue or shRNA suppression of b-catenin (n=15/condition). **e**, Kaplan-Meier survival curve for CH-157MN xenograft overall survival in NU/NU mice as in **d** (n=15/condition, log-rank test). **f**, Normalized ADC from MRI of CH-157MN xenografts as in **d** and **e**. **g**, Model of meningioma Wnt signaling in the context of Merlin post-translational modifications, PP1A and PKC activity, cancer-associated missense mutants, and meningioma ADC. Lines represent means, and error bar represent standard error of the means. *P≤0.05, **P≤0.01, ***P≤0.0001 (Student’s t-test, one sided).

To study associations between meningioma ADC and tumor biology, 100 preoperative MRIs from meningiomas with available DNA methylation profiling and targeted exome sequencing of the *NF2* locus were retrospectively reviewed and imaging features were analyzed in the context of clinical follow-up data^7^. Meningioma ADC was dichotomized at the mean (Fig. 4a), and ADC high meningiomas had favorable clinical outcomes compared to ADC low meningiomas (5-year local freedom from recurrence 90.3% versus 48.7%, p < 0.0001, log-rank test) (Fig. 4b). DNA methylation profiling controlled for artifacts from chromosome copy number variants (CNVs) reveals meningiomas are comprised of Merlin-intact, Immune-enriched, and Hypermitotic DNA methylation groups^7^, which are concordant with groups and subgroups of meningiomas derived from RNA sequencing or DNA methylation profiling integrated with RNA sequencing, CNVs, and SSVs^8,11,12^. Analysis of DNA methylation groups across ADC high versus ADC low meningiomas showed the majority of ADC high meningiomas were Merlin-intact (56% versus 16%, p < 0.0001, chi-squared test) (Fig. 4c). To determine if Wnt signaling underlies meningioma ADC, β-catenin was suppressed in meningioma cells using shRNAs (Extended Data Fig. 7a), which inhibited Wnt signaling (Extended Data Fig. 7b), attenuated cell proliferation and tumor growth (Fig. 4d and Extended Data Fig. 7c), and prolonged overall survival compared to meningioma cells and xenografts expressing shNTC (Fig. 4e). MRI of Merlin-deficient meningioma xenografts showed Merlin^WT^ or Merlin^S13A^ rescue did not alter ADC, but ADC was increased with Merlin^S13D^ rescue or Merlin^WT^ rescue with concurrent suppression of β-catenin (Fig. 4f). Thus, meningioma ADC is inversely correlated with Wnt pathway activation, and ADC high meningiomas have favorable clinical outcomes in human patients and preclinical xenograft models.

## Discussion

Flere we show Merlin drives meningioma Wnt signaling and tumor growth through a feed-forward mechanism that requires Merlin^S13^ dephosphorylation to attenuate inhibitory interactions with β-catenin and activate the Wnt pathway (Fig. 4g). Integrating data from meningioma xenografts and patients, our results establish meningioma ADC as a potential non-invasive imaging biomarker of Wnt signaling in Merlin-intact meningiomas with S13 phosphorylation and favorable clinical outcomes (Fig. 4g). These data shed new light on how meningiomas can grow despite expressing the canonical tumor suppressor *NF2*/Merlin. Nevertheless, Merlin-intact meningiomas tend to be benign and have the most favorable outcomes across molecular groups of human meningiomas^7,8,11,12^. Many meningiomas can be safely observed with serial imaging surveillance^13^, but differentiating benign from aggressive meningiomas without subjecting patients to the morbidities associated with potentially unnecessary surgical treatments has been a barrier to improving clinical paradigms for patients with the most common primary intracranial tumor. To address this unmet need for patients with meningiomas, our data elucidate a non-invasive, clinically-tractable MRI biomarker that sheds light on meningioma biology and could be used to guide treatment de-escalation or imaging surveillance.

Our finding that Merlin post-translational modification can promote oncogenic Wnt signaling is unexpected considering the myriad tumor suppressor functions associated with *NF2*. CNVs deleting *NF2* on chromosome 22q are early events underlying Immune-enriched or Hypermitotic meningioma tumorigenesis^7,27^, but Merlin-intact meningiomas encode SSVs targeting *TRAF7, AKT1,* or *KLF4* that may be tumor-initiating^16,17^. Our data suggest Merlin^S13^ phosphorylation status and Wnt signaling may modify Merlin-intact meningioma growth. Proximity-labeling proteomic mass spectrometry coupled with mechanistic and functional approaches demonstrate PKC and PP1A are important for this signaling mechanism, but other (1) kinases or phosphatases, (2) Merlin domains, or (3) Wnt pathway members may also contribute to meningioma Wnt signaling. In support of that hypothesis, we show (1) suppression of PP1A or PKC isoforms partially regulates Merlin^S13^ phosphorylation (Fig. 3i, j), (2) epistatic Merlin^S13D^ or Merlin^ΔNTD^ rescue partially restores Wnt signaling in meningioma cells (Fig. 3c, e), and (3) the non-canonical Wnt pathway regulators AMOT, AMOTL1, AMOTL2, DSG2, and DLG1 were identified in proximity to Merlin alongside β-catenin in meningioma cells^38-40^ (Supplementary Table 2). Indeed, the Wnt pathway can be activated by multiple mechanisms in meningiomas with unfavorable clinical outcomes^23^, and not all Merlin-intact meningiomas can be controlled with existing therapies of surgery or radiotherapy^7,12^. Thus, our results also shed light on potential targets for future molecular therapies that could be used to treat meningiomas which are resistant to standard interventions.

To our knowledge, the Merlin^S13^ phosphorylation site that sequesters β-catenin to the plasma membrane and inhibits the Wnt pathway has not been previously reported. The NTD in Merlin is unique compared to other FERM family members but is evolutionarily conserved across Merlin orthologs in higher eukaryotes. Previous studies show Merlin serine 518 (S518) phosphorylation regulates tumor suppressor functions by controlling protein conformation^41-44^, but cellular mechanisms that are influenced by Merlin^S518^ phosphorylation are incompletely understood and how tertiary conformational changes affect Merlin functions are unclear. Like other FERM family members, the open/closed conformation of Merlin requires interactions between the CTD and the FERM domain^43,45,46^, which are not influenced by S13 phosphorylation on the flexible NTD (Fig. 3a). Consequently, our data reveal novel structural/functional insights for an important and extensively studied tumor suppressor.

## Methods

This study complied with all relevant ethical regulations and was approved by the UCSF Institutional Review Board (IRB #10-01141 and #18-24633). As part of routine clinical practice at both institutions, all patients who were included in this study signed a waiver of informed consent to contribute data and tissue to research.

### Cloning

Plasmids encoding genes of interest were purchased from Addgene, or when unavailable, PCR amplified from cDNA. PCR products were cut using sticky-end restriction enzymes and ligated into plasmids with T4 ligase (NEB, Cat# M0202L). Ligated plasmids were transformed into Top10 or Stable II *E.coli,* colonies were isolated and expanded, and plasmid DNAs were sent for Sanger sequencing to confirm genes of interest.

### Cell culture

IOMM-Lee, CH-157MN, HEI-193, and HEK293T cells were purchased from ATCC and cultured in Dulbeco’s Modified Eagle’s Medium (DMEM) (Gibco, Cat# 11960069) supplemented with 10% Fetal Bovine Serum (FBS) (Gibco, Cat# 26140-079) and 1X GlutaMAX (Gibco, Cat# 35050079). M10G cells were isolated from a primary human meningioma as previously reported^47^ and cultured in 1:1 growth media of DMEM F12 (Fisher Scientific, Cat# 11-320-082) and Neurobasal media (Gibco, Cat# 10888022) supplemented with 5% FBS, 1X GlutaMAX, 1mM NEAA (Gibco, Cat# 11140050), N2 supplement (Gemini Bio-Products, Cat# 400-163), B27 (Gibco, Cat# 12587-010), 20ng/ml epidermal growth factor (PeproTech, Cat# AF-100-15), and 20ng/ml fibroblast growth factor (PeproTech, Cat# AF-100-18B).

### Plasmid overexpression

Cells were seeded into 6cm plates at 0.5x10^5^ cells/ml for 24 hours before transfection. Transfection solution consisting of 500ml Opti-MEM (Thermo Fisher Scientific, Cat# 51985091, 2mg plasmid DNA and 7.5ml FuGene (Promega, Cat# E2311) was incubated at room temperature for 20 minutes before adding to cells. Media was changed the following day and cells were harvested 48-hours after transfection for experimentation.

### siRNA knockdown

On day 1, reverse transfection of siRNA was achieved using RNAi-MAX (Invitrogen, Cat# 13778075). For each 6cm plate, solution A (250ml Opti-MEM, 25nM siRNA) and solution B (250ml Opti-MEM and 5ml RNAiMAX) were made independently. The solutions were incubated at room temperature for 5 minutes and then combined, vortexed, and incubated for an additional 20 minutes before adding to cells at 0.2 x 10^5^ cells/ml for seeding. Media was changed on day 2. RNAi-Max knockdown was repeated on day 3 followed by media change on day 4. For experiments which included plasmid overexpression, Fugene transfection and simultaneous reseeding of cells was performed on day 5 for 48 hours. Cells were harvested 7 days after the first siRNA transfection for experimentation.

### Lentiviral Transduction

For virus production, HEK293T cells were seeded at 2x10^5^ cells/ml in 10cm plates. Lentiviral packaging plasmids pMD2.G and psPAX2 were transfected using the Mirus Trans-IT (Mirus Bio, Cat# MIR 2225) virus transfection reagent. For transduction, HEK293T viral media was filtered through a 0.45mm syringe filter and placed over host cells with 1:1 host cell media and 10nM polybrene (EMD Millipore, Cat# TR-1003-G). After 48 hours, cells were treated with blastocydin (Goldbio, Cat# B-800-25) or puromycin (Invivogen, Cat# ant-pr-1) for antibiotic selection of stably expressing cells.

### Immunoblotting

Cells were lysed in either RIPA (150mM sodium chloride, 50mM Tris-HCl [pH8], 1 % NP40, 0.25% sodium deoxycholate, 0.1% SDS) or JIES buffer (100mM NaCl2, 20mM Tris-HCl [pH7.4], 5mM MgCl2, 0.5% NP40) containing protease inhibitor (Complete-Mini, Sigma Aldrich, Cat# 11836170001) and phosphatase inhibitor (Phos-STOR Roche, Cat# 04906837001) and quantified using the Bradford assay (Bio-Rad, Cat# 5000205). Normalized protein lysates were run on 4-20% TRX gels (Bio-Rad, Cat# 4561096), transferred onto nitrocellulose membranes (Bio-Rad, Cat# 1620094), blocked in 5% BSA in TBST, probed with primary antibodies at the indicated concentrations, and incubated with HRP-conjugated secondary antibodies (1:2000 dilution). Primary and secondary antibodies used were FLAG (Sigma-Aldrich, Cat# F7425, 1:500-2000), DYKDDDDK (Cell Signaling, Cat# 14793S, 1:2000), HA (Cell Signaling, Cat# 2999S, 1:2000), GAPDH (Thermo Fisher Scientific, Cat# MA515738, 1:5000), Merlin (Abcam, Cat# ab88957, 1:1000), α-Tubulin (Sigma-Aldrich, Cat# T5168, 1:5000), calreticulin (Abcam, Cat# ab92516, 1:10,000), vimentin (Abcam, Cat# ab8069, 1:10,000), Rb (Cell Signaling, Cat# 9309S, 1:5000), Histone H3 (Thermo, Cat# 702023, 1:5000), b-catenin (BD Biosciences, Cat# 610153, 1:1000). Anti-phospho-Serine13 (1:500) was a custom antibody developed by Thermo Fisher Scientific using rabbits that were immunized with a synthetic Merlin phospho-peptide sequence (CSRMSFS(pS)LKRKQP-amide). Chemiluminescence was detected with Pierce ECL (Thermo Scientific, Cat# 32209) and developed on autoradiography film after incubation with rabbit (Cell signaling, Cat# 7074, 1:2000) or mouse (Cell signaling, Cat# 7076, 1:2000) HRP-conjugated secondary antibodies.

### Immunofluorescence and confocal microscopy

Cells were seeded onto glass coverslips in 24-well plates. 24 hours prior to fixing, cells were treated with 0.1mg/ml doxycycline and APEX labelled as described below. Quenched cells were fixed in PFA (Electron Microscopy Sciences, Cat# 15710) and stained using DYKDDDDK primary antibody (Cell Signaling, Cat# 14793S, 1:2000) and anti-mouse conjugated to Alexa Fluor 488 secondary antibody (Thermo Fisher Scientific, Cat# A21202, 1:2000), streptavidin conjugated to Alexa Fluor 647 (Thermo Fisher Scientific, Cat# S21374, 1:1000), and Hoechst (Invitrogen, Cat# H3570, 1:10,000). Coverslips were mounted onto slides using Prolong Diamond anti-fade mounting media (Thermo Fisher Scientific, Cat # P36965) and imaged on a Zeiss LSM800 fluorescence microscope.

### Immunohistochemistry and light microscopy

Deparaffinization and rehydration of 5 μm FFPE meningioma xenograft sections and hematoxylin and eosin staining were performed using standard procedures. Immunostaining was also performed on 5 μm FFPE meningioma xenograft sections using an automated Ventana Discovery Ultra staining system. Immunohistochemistry was performed using rabbit monoclonal Ki-67 (Ventana, clone 30-9, Cat# 790-4286, 1:6) with incubation for 16 min. Histologic and immunohistochemical features were evaluated using light microscopy on an BX43 microscope with standard objectives (Olympus). Images were obtained and analyzed using the Olympus cellSens Standard Imaging Software package.

### Immunoprecipitation

After protein lysis and quantification, protein content was normalized across samples and incubated with pre-washed HA (Sigma-Aldrich, Cat# A2095, 50ml per IP) or FLAG (Sigma-Aldrich, Cat# M8823, 50ml per IP) antibody-bound beads. The sample/bead slurry was left to rotate at 4°C for 4 hours before washing 4 times in lysis buffer supplemented with protease and phosphatase inhibitors as described above. Bound proteins were eluted from beads by boiling in 2x Laemmli buffer and separated by gel electrophoresis as described above.

### Luciferase assay

Cells were transfected with pRL-TKTOP-Flash Tcf/Lef luciferase reporter and with or without additional genes of interest, as indicated, for 48 hours. 24 hours before experimentation, cells were treated with or without 200ng/ml Wnt3a (R&D Systems, Cat# 5036-WN). Luciferase activity was detected using the Dual Luciferase Kit (Promega, Cat# G4100) and a GLO-Max Promega plate reader.

### QPCR

QPCR primers were designed using PrimerBank (https://pga.mgh.harvard.edu/primerbank/). RNA was isolated using the QiaGen RNeasy prep kit (Cat# 74106). cDNA was prepared using 1000ng of RNA and iScript reverse transcriptase (Bio-Rad, Cat# 1708891). QPCR was performed using PowerUp SYBR Green (Thermo Scientific, Cat# A25742) in a Life Technologies QuantStudio 6 Flex Real Time PCR system. Relative gene expression was calculated using the DDCt method against a control gene, *GAPDH*. QPCR primers used were NF2_F(5’-TTGCGAGATGAAGTGGAAAGG-3’), NF2-R (5’-CAAGAAGTGAAAGGTGACTGGTT-3’), PP1A_F (5’-ACTACGACCTTCTGCGACTAT-3’), PP1A_R (5’-AGTTCTCGGGGTACTTGATCTT-3’), PRKCA_F (5’-GTCCACAAGAGGTGCCATGAA-3’), PRKCA_R (5’-AAGGTGGGGCTTCCGTAAGT-3’), PRKCG_F (5’-AGCCACAAGTTCACCGCTC-3’), PRKCG_R (5’-GGACACTCGAAGGTCACAAAT-3’), CTNNB1_F (5’-CATCTACACAGTTTGATGCTGCT-3’), CTNNB1_R (5’-GCAGTTTTGTCAGTTCAGGGA-3’), GAPDH_F (5’-GTCTCCTCTGACTTCAACAGCG-3’), and GAPDH_R (5’-ACCACCCTGTTGCTGTAGCCAA-3’).

### Proximity-labeling proteomic mass spectrometry

M10G cells stably expressing pLV.APEX2 constructs encoding wildtype or variant Merlin constructs were seeded onto 5 x 15cm plates. Cells were treated with 0.1mg/ml doxycycline to induce Merlin expression 24 hours before APEX labelling. For labelling, 0.5mM biotin-phenol (Berry and Associates, Cat# BT1015) was added to each plate for 30 minutes at 37°C, and 1mM H_2_O_2_ (Sigma Aldrich, Cat# H1009) was added to cell media on ice for 30 seconds to initiate the reaction. Media was replaced with quenching media (10mM Sodium Ascorbate, 1mM Azide, 5mM Trolox) for 2 washes. For mass spectrometry, cells were scraped and pelleted for biotin/streptavidin precipitation as previously described^7^.

### Mouse xenografts

ForshRNA induction, cells were pre-treated with 3mM IPTG (Sigma Aldrich, Cat# I6758) 10-days prior to injection. Xenograft experiments were performed by injecting 3 million human meningiomas cells, either CH157-MN or IOMM-Lee, into the flank of 4–6-week-old NU/NU female mice (Envidigo). To induce plasmid or shRNA expression, mice were treated with or without 200mg/ml doxycycline (Sigma-Aldrich, Cat# D9891) and with or without 10M IPTG in cage water that was changed every 2-3 days. Kaplan-Meyer curves were created by recording deaths at the protocol end of 50% ulcerated tumor or tumor >2000 mm^3^. Tumors were processed for single-cell dissociation and single-cell RNA sequencing, QPCR, or immunoblotting.

### Xenograft lysis

Tumors were dissected into small chunks and allocated for either RNA extraction for cDNA synthesis and QPCR, or protein extraction and immunoblotting. Tumor chunks were placed in a 2ml Eppendorf tube with a 7mm metal bead and 350ml of RLT lysis buffer for RNA extraction, or 300ml RIPA buffer for protein. Tubes were shaken in a TissueLyser for 2 minutes at 30mhz, lysate was cleared by spinning in a desktop centrifuge at full speed for 5 minutes at 4°C and used for downstream analysis.

### Single-cell dissociation of solid tumors

Tumors were freshly harvested from mice and sliced into small pieces using two #10 scalpels before enzymatic dissociation in 0.1 mg/ml Colagenase Type 7 (Worthington Biochemicals, Cat# LS005332) at 37°C for 30 minutes, followed by 0.25% trypsin (Thermo Fisher Scientific, Cat# 25200114) digest at 37°C for 10 minutes. Red blood cells were removed in 1X RBC lysis buffer (Invitrogen, Cat# 00-4300-54) at room temperature for 10 minutes. Sequential filtering of cells through 70mm and 40mm filters and cell counting using a Life Technologies Countess II was performed to generate a single-cell suspension.

### Single-cell RNA sequencing and analysis

Single cells were processed through a 10X Genomics Chromium Controller and libraries were created using the Chromium Single Cell 3’ Library & Gel Bead Kit v3 (10X Genomics, Cat# 1000121) according to the manufacturers protocol with an intended yield of 8,000 single cells. Libraries were sequences on a NovaSeq S4 6000 at the UCSF Center for Advanced Technology.

Demultiplexing, identification of empty droplets, removal of duplicates, and alignment to the human or mouse reference genomes was performed using the CellRanger v6.1.2 pipeline. Count matrices were selected using cells with more than 50 unique genes, less than 8000 unique genes, and with less than 20% of genes assigned as mitochondrial. Data were processed using Seurat in RStudio using SCTransform. Dimensionality reduction was performed using principal component analysis and uniform manifold approximations and projections (UMAP) were performed on the reduced dimensionality data using the minimum distance of 0.2 and a Louvain clustering resolution of 0.8. Differentially expressed genes were identified with Wilcoxon Rank Sum test in Seurat.

### Structural modeling

To obtain a full-length 3D representation of the Merlin human protein, a structural model was generated using the Robetta server (https://robetta.bakerlab.org/) and the solved structure of human Merlin-FERM as a template (PDB 4ZRJ). The model was inspected using pymol (v2.x) visualization software to rationalize the role of disease-associated mutations. Robetta provides a fully automated modeling procedure exploiting both *ab initio* and comparative models of protein domains. Comparative models were built from structures detected and aligned by HHSEARCH, SPARKS, and Raptor. Loop regions were assembled from fragments and optimized to fit the aligned template structures.

### Human magnetic resonance imaging

All patients underwent MRI examinations on a 3T Discovery MR750 scanner (GE Healthcare) using an eight-channel phased-array head coil prior to surgical resection. The imaging protocol included anatomical T2-weighted Fluid Attenuated Inversion Recovery (FLAIR) and Fast Spin Echo (FSE) images, along with 3D T1-weighted Inversion Recovery-Spoiled Gradient Recalled echo (IR-SPGR) imaging pre- and post-injection of a gadolinium-based contrast agent. Diffusion-weighted imaging or DTI was obtained in the axial plane with b=1000s/mm^2^ and either 6 gradient directions and 4 excitations or 24 gradient directions and 1 excitation or b=2000s/mm^2^ and 55 gradient directions (echo time/repetition time=108/1000ms, voxel size=1.7-2.0x1.7-2.0x2.0-3.0mm). To calculate ADC maps, a pipeline using the FMRIB’s Diffusion Toolkit was applied to the diffusion-weighted imaging and DTI data as previously described^48^.

### Preclinical (mouse xenograft) magnetic resonance imaging

*In vivo* MRI was performed on a Cryogen free 3T Bruker Biospin (Billerica) with a maximum gradient strength of 960mT/m and a maximum slew rate of 3550 T/m/s. Multi-slice T2-weighted images were acquired using a Rapid Acquisition with Relaxation Enhancement (RARE) sequence with the following parameters: echo time/repetition time=48/4000ms, RARE-factor=8,4 signal averages, field of view=32x32mm, 25 slices with 1.0mm slice thickness, in-plane resolution of 0.167x0.167mm, resulting in an imaging time of 6 minutes and 24 seconds.

Multi-slice diffusion tensor imaging (DTI) was acquired using a single-element diffusion-weighted echo-planar imaging sequence with the following parameters: echo time/repetition time=30/2500ms, 8 signal averages, 3 diffusion directions, two b-values per direction (b–500 and 1000s/mm^2^), in-plane resolution of 0.333x0.333mm with a partial Fourier factor of 1.5 in the phase-encoding direction, and the same field of view, slice thickness, and slice number as the T2-weighted images. With respiratory gating, the total imaging time was 2 minutes and 20 seconds. To generate apparent diffusion coefficient (ADC) maps, Horos (Lesser General Public License, v3.0) imaging software was used to manually place polygonal regions-of-interest (ROIs) over the solid portion of the meningioma xenograft tumors and these volume measurements were averaged for each group. Areas of cystic change or hemorrhage as denoted by T2-weighted images were excluded.

### Statistics

No statistical methods were used to predetermine sample sizes, but our cohort sizes are similar or larger to those reported in previous publications. Data distribution was assumed to be normal, but this was not formally tested. Investigators were blinded to conditions during clinical data collection and analysis. Bioinformatic analyses were performed blind to molecular characteristics. The clinical samples used in this study were non-randomized with no intervention, and all samples were interrogated equally. Thus, controlling for covariates among clinical samples is not relevant. Unless specified otherwise, lines represent means, and error bars represent standard error of the means. Results were compared using Student’s t tests, Chi-squared tests, and log-rank tests, which are indicated in the text, methods, and figure legends alongside approaches used to adjust for multiple comparisons. Statistical significance is shown by *p£0.05, **p£0.01, or ***p£0.0001.

## Data Availability

Single-cell RNA sequencing data (n=12 human meningioma xenograft samples) reported in this manuscript have been deposited in the NCBI Gene Expression Omnibus under the accession GSE224347. Transcriptomes were simultaneously aligned against publicly available hg19 and mm10 data sets, stored on the UCSF C4 environment at refdata-cellranger-hg19-and-mm10-3.0.0/refdata-cellranger-mm10-3.0.0/. Cells with transcripts aligned to <99% of the human dataset were discarded.

## Code availability

The open-source software, tools, and packages used for data analysis in this study, as well as the version of each program, were cellRanger (v6.1.2), R (v4.2.1), Seurat R package (v4.2.1), SCTransform (0.3.5), Harmony R package (v0.1.0), and Horos (v3.0). No software was used for data collection. Code is available on github (www.github.com/cdeaton380/Merlin-rescue.git).
